# HIV-1 transgene expression in rats causes oxidant stress and alveolar epithelial barrier dysfunction

**DOI:** 10.1186/1742-6405-6-1

**Published:** 2009-02-04

**Authors:** Coy Lassiter, Xian Fan, Pratibha C Joshi, Barbara A Jacob, Roy L Sutliff, Dean P Jones, Michael Koval, David M Guidot

**Affiliations:** 1 Department of Medicine, Division of Pulmonary, Allergy and Critical Care Medicine, Emory University School of Medicine, Atlanta, Georgia, USA; 2 The Section of Pulmonary and Critical Care Medicine, Atlanta VA Medical Center, Decatur, Georgia, USA

## Abstract

**Background:**

HIV-infected individuals are at increased risk for acute and chronic airway disease even though there is no evidence that the virus can infect the lung epithelium. Although HIV-related proteins including gp120 and Tat can directly cause oxidant stress and cellular dysfunction, their effects in the lung are unknown. The goal of this study was to determine the effects of HIV-1 transgene expression in rats on alveolar epithelial barrier function. Alveolar epithelial barrier function was assessed by determining lung liquid clearance *in vivo *and alveolar epithelial monolayer permeability *in vitro*. Oxidant stress in the alveolar space was determined by measuring the glutathione redox couple by high performance liquid chromatography, and the expression and membrane localization of key tight junction proteins were assessed. Finally, the direct effects of the HIV-related proteins gp120 and Tat on alveolar epithelial barrier formation and tight junction protein expression were determined.

**Results:**

HIV-1 transgene expression caused oxidant stress within the alveolar space and impaired epithelial barrier function even though there was no evidence of overt inflammation within the airways. The expression and membrane localization of the tight junction proteins zonula occludens-1 and occludin were decreased in alveolar epithelial cells from HIV-1 transgenic rats. Further, treating alveolar epithelial monolayers from wild type rats *in vitro *with recombinant gp120 or Tat for 24 hours reproduced many of the effects on zonula occludens-1 and occludin expression and membrane localization.

**Conclusion:**

Taken together, these data indicate that HIV-related proteins cause oxidant stress and alter the expression of critical tight junction proteins in the alveolar epithelium, resulting in barrier dysfunction.

## Background

Individuals infected with the human immunodeficiency virus (HIV) are susceptible to both routine and opportunistic infections of the lung. Despite the advent of highly active anti-retroviral therapy (HAART), lung disease continues to be the leading cause of death [1,2]. Interestingly, although infections account for the apparent majority of these lung-related deaths, there is growing evidence that HIV also increases the risk of chronic airway diseases such as emphysema. For example, an analysis of the Veterans Aging Cohort Study showed that HIV-infected subjects were 50–60% more likely to develop chronic obstructive pulmonary disease (emphysema and/or chronic bronchitis) than HIV-negative subjects, and that HIV infection was an independent risk factor even after accounting for age, race/ethnicity, smoking histories, and substance abuse [3]. Other studies show similar results and several recent excellent reviews summarize the clinical data to date as well as some of the laboratory-based research that is beginning to explore possible mechanisms [4-6]. A common target in both acute lung injuries such as pneumonia and chronic lung diseases such as emphysema is alveolar epithelial damage. However, whether or not chronic HIV infection renders the alveolar epithelium susceptible to injury is unknown.

There is abundant evidence that oxidant stress is associated with a wide range of lung diseases [7,8]. Within the alveolar space, the thiol antioxidant glutathione (GSH) plays a key role in detoxifying endogenous and exogenous oxidants and limits the oxidation of cysteine residues in many proteins, thereby regulating redox signaling [9,10]. The preservation of an adequate GSH pool within the alveolar space is therefore critical to control cell signaling, the transcription of pro-inflammatory genes, cell proliferation and survival [7,8,11]. Unfortunately, HIV infection is associated with significant oxidant stress and decreased systemic levels of GSH [12-14]. Within the lung, there is evidence that GSH levels decline as the disease progresses, whereas the lungs of asymptomatic individuals have preserved levels of GSH [15,16].

Importantly, not all of the consequences of HIV infection can be attributed solely to direct viral infection. It has been reported that HIV-related proteins released by infected cells can enter the central nervous system (CNS) from the blood and affect CNS function independently of viral transport [17]. For example, the HIV envelope protein, gp120, and the transregulatory protein Tat, can stimulate endothelial cells to secrete neuro-immunoactive substances [17]. It is interesting to note that gp120 and Tat can directly induce oxidant stress and GSH depletion in isolated endothelial cells, and this appears to contribute to tight junction disruption and compromise of the blood-brain barrier [18,19]. Although the mechanisms by which these HIV-related proteins cause oxidant stress are not completely understood, it has been shown that Tat can repress the expression of the mitochondrial form of superoxide dismutase in HeLa cells [20]. Whether this repression of superoxide dismutase or some other oxidant stress induced by these proteins decreases the intracellular GSH pool is unclear. Transgenic animal studies extend such *in vitro *studies and provide perhaps the most compelling evidence implicating HIV-related proteins in the pathophysiology of the disease [21]. Reid and colleagues established a non-infectious HIV-1 transgenic (HIV-1 Tg) rat model that expresses an HIV-1 provirus regulated by the viral promoter but with a functional deletion of *gag and pol *[22,23]. This HIV-1 Tg rat develops a progressive AIDS-like phenotype as it ages including immunologic dysfunction, nephropathy, muscle wasting, skin lesions and cataracts [22,23]. We recently reported that these HIV-1 transgenic rats have soluble gp120 in their alveolar epithelial lining fluid and have significant defects in alveolar macrophage immune function [10]. These findings were provocative as they suggest that the well-known effects of HIV infection on this resident immune cell within the alveolar space may not be entirely due to viral infection and replication *per se*, but that HIV-related proteins could exert pathophysiological effects within this microenvironment. Further, although the effects of HIV infection on alveolar macrophage function have been studied extensively, very little attention has focused on possible effects on the alveolar epithelium as this cell type is not infected by the virus. However, as HIV-related proteins are present in the alveolar space during chronic infection and have toxic effects on other cell types that are likewise not infected directly by the virus, and because HIV-infected individuals are also susceptible to diseases associated with alveolar epithelial dysfunction, we investigated the effects of HIV-1 transgene expression on alveolar epithelial function in the rat model. In this study, we report that chronic expression of HIV-related proteins *in vivo *causes profound oxidant stress and GSH depletion in the lung. In parallel, these HIV-related proteins cause significant alveolar epithelial barrier dysfunction that is associated with changes in tight junction proteins that can be reproduced in primary alveolar epithelial cells *in vitro *by direct exposure to gp120 and Tat. Taken together, these results provide the first evidence that HIV-related proteins affect alveolar epithelial barrier function and could thereby render infected individuals susceptible to acute or chronic causes of respiratory failure.

## Results

### HIV-1 transgene expression caused significant oxidant stress in the lung

**Figure 1:**
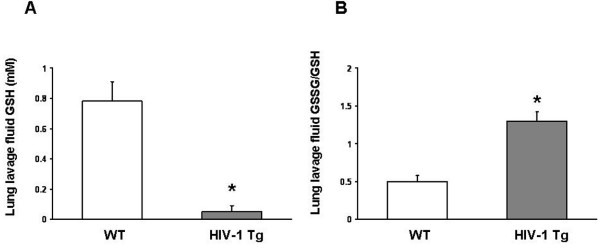
**HIV-1 transgene expression decreased glutathione levels and altered the glutathione redox balance in the alveolar space**. Lung lavage fluid from HIV-1 transgenic (HIV-1 Tg) rats had significantly decreased (* P = 0.0016) levels of glutathione (GSH) as shown in panel A, and significantly increased (* P = 0.0049) ratios of glutathione disulfide (GSSG; the predominant form of oxidized glutathione) to GSH as shown in panel B, when compared to lung lavage fluid from wild type (WT) rats. The levels of GSH and GSSG were determined by HPLC as described in the Methods. Each value represents the mean ± SEM of 4 rats.

**Figure 2:**
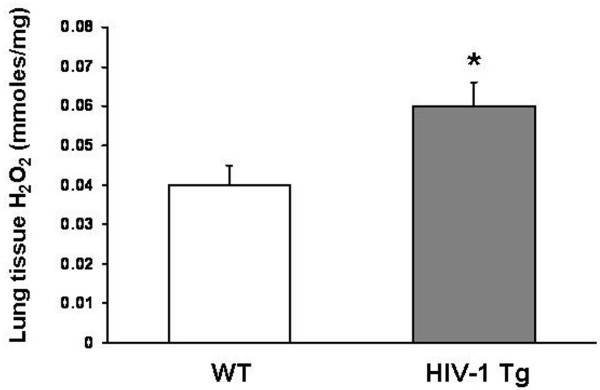
**HIV-1 transgene expression increased the levels of hydrogen peroxide levels in lung tissue**. Lung tissue from HIV-1 transgenic (HIV-1 Tg) rats had significantly increased (* P < 0.0001) levels of hydrogen peroxide (H_2_O_2_) compared to lung tissue from wild type (WT rats). H_2_O_2 _levels were determined by the Amplex Red fluorescent detection technique as described in the Methods. Each value represents the mean ± SEM of 6 rats.

We first determined the relative levels of glutathione (GSH) and its primary oxidized form, glutathione disulfide (GSSG) in the lung lavage fluids of wild type (WT) and HIV-1 transgenic (HIV-1 Tg) rats. As shown in Figure 1, panel A, GSH levels were decreased >90% (P = 0.0016) in HIV-1 Tg rats compared to WT rats. In parallel, there was evidence of significant GSH oxidation; specifically, and as shown in Figure 1, panel B, the relative levels of glutathione disulfide (GSSG; the predominant form of oxidized glutathione) to GSH, expressed as the GSSG:GSH ratio (a commonly used index of oxidant stress), were increased nearly 3-fold (P = 0.0049) in HIV-1 Tg rats compared to WT rats. Further evidence of oxidant stress in the lungs of HIV-1 Tg rats is shown in Figure 2. Lung tissue levels of hydrogen peroxide were significantly increased (P < 0.0001) in HIV-1 Tg rats compared to WT rats. In contrast, despite the oxidant stress within the alveolar space of HIV-1 transgenic rats, there was no evidence of significant inflammation. Specifically, the cell counts and differentials were not different (P > 0.05) in the lung lavage fluids of HIV-1 transgenic rats when compared to wild type rats (Table 1; shown are the mean values ± SEM of 4 rats in each group; for the differential counts, an average of 525 cells per lavage fluid sample were analyzed. In parallel, the levels of interleukin-2 (IL-2), tumor necrosis factor-α (TNFα) and interleukin-4 (IL-4) were the same (P > 0.05) in the lung lavage fluids of HIV-1 transgenic rats when compared to wild type rats (Table 2; shown are the mean values ± SEM of 4 rats in each group; note the trend toward an increase in IL-2 in the HIV-1 transgenic rats but with significant variation in the levels of this cytokine). Although these latter results do not exclude perturbations in the control of inflammation within the alveolar space, they nevertheless suggest that chronic expression of HIV-1-related proteins causes significant oxidant stress but this is not accompanied by overt chronic inflammation. This is consistent with our previous findings that HIV-1 transgenic expression dampens the immune functions of the resident alveolar macrophage [10]. These findings are also consistent with the effects of chronic alcohol ingestion on the lung, which causes significant glutathione depletion within the alveolar space but does not by itself cause overt lung inflammation or injury [24-26].

**Table 1:** Comparison of total cells and differential cell counts from rat BAL between wild type and HIV-1 transgenic rats.

**Cell type**				
***Group***	Total cells (×10^6^)	Macrophages (%)	Lymphocytes (%)	Neutrophils (%)

***Wild Type***	1.96 ± 0.2	97.92 ± 0.7	1.61 ± 0.5	0.47 ± 0.2

***HIV-1 Tg***	2.36 ± 0.1	97.27 ± 0.2	1.90 ± 0.2	0.82 ± 0.2

**Table 2:** Comparison of pro- and anti-inflammatory cytokines from BAL in wild type and HIV-1 transgenic rats.

	**Wild Type**	**HIV-1 Tg**
IL-2 (pg/ml)	20.2 ± 10.8	98.5 ± 58.9

TNFα (pg/ml)	0.5 ± 0.2	1.1 ± 0.3

IL-4 (pg/ml)	4.4 ± 1.1	6.4 ± 1.2

### HIV-1 transgene expression impaired alveolar epithelial barrier function

**Figure 3:**
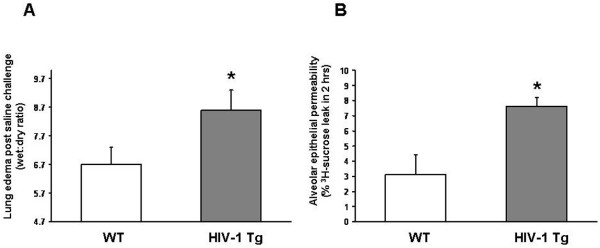
**HIV-1 transgene expression impaired alveolar epithelial barrier function *in vivo *and increased permeability of alveolar epithelial monolayers *in vitro***. Panel A shows the relative lung liquid clearance, as reflected by lung tissue wet:dry ratios 30 min following intratracheal challenge with 2 cc of saline (see Methods for details), in wild type (WT) vs. HIV-1 transgenic (HIV-1 Tg) rats. The wet:dry ratios in HIV-1 Tg rats were increased ~2-fold higher above baseline than WT rats (* P < 0.05), reflecting a significantly decreased ability to clear saline from the lung. Note that the baseline wet:dry ratio of the rat lung is 4.5, and therefore the data are plotted to reflect the relative increases from this baseline in each group. Each value represents the mean ± SEM of 6 rats. Panel B shows the relative paracellular permeability of alveolar epithelial monolayers derived from WT vs. HIV-1 TG rats, as reflected by the flux of ^3^H-sucrose across each monolayer in 2 hrs (see Methods for details). Alveolar epithelial monolayers derived from HIV-1 Tg rats had an ~3-fold increase (* P < 0.05) in paracellular permeability when compared to alveolar epithelial monolayers derived from WT rats. Each value represents the mean ± SEM of alveolar epithelial monolayers from 6 rats.

To investigate epithelial barrier function *in vivo*, we examined lung liquid clearance following intra-tracheal saline challenge as we have previously described [27,28]. This technique provides a sensitive index of overall lung epithelial barrier function, which is the integration of active fluid transport and paracellular permeability. As shown in Figure 3, panel A, wet:dry ratios in HIV-1 Tg rats following saline challenge were increased ~2-fold higher above baseline than comparably challenged WT rats (P < 0.05), reflecting a significantly decreased ability to clear saline from the lung. Note that the baseline wet:dry ratio of the rat lung is 4.7 as we have published previously [27], and therefore the data are plotted to reflect the relative increases from this baseline in each group. As decreased lung liquid clearance can reflect either an increase in epithelial paracellular permeability or a decrease in transcellular fluid transport, we next determined alveolar epithelial paracellular permeability *in vitro*. We have used and published this combination of lung liquid clearance *in vivo *and alveolar epithelial paracellular permeability *in vitro *to examine the effects of chronic alcohol ingestion on the alveolar epithelial barrier [27,28]. As shown in Figure 3, panel B, alveolar epithelial monolayers derived from HIV Tg rats had an ~3-fold increase (P < 0.05) in paracellular permeability, as reflected by ^3^H-sucrose flux in 2 hrs, when compared to alveolar epithelial monolayers derived from WT rats. Therefore, the defect in lung liquid clearance shown in Figure 3, panel A appears to be due at least in part to a relative increase in alveolar epithelial permeability induced by HIV-1 transgene expression. Importantly, these effects on the alveolar epithelial barrier could not be explained by transgene expression of HIV-related proteins within the alveolar epithelium *per se*, as gp120 expression was not detected in these cells by ELISA (not shown), even though gp120 protein was easily detectable in even dilute lung lavage fluid in our previous study [10]. This is consistent with the clinical scenario, where HIV does not infect the lung epithelium but can be found along with its related proteins within the alveolar space [29].

### HIV-1 transgene expression was associated with alterations in the tight junction proteins zonula occludens-1 and occluding

**Figure 4:**
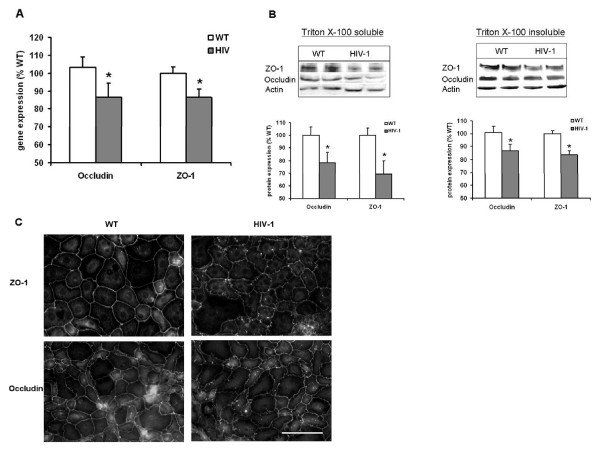
**HIV-1 transgene expression altered the expression of two key components of alveolar epithelial tight junctions, occludin and zonula occludens-1 (ZO-1)**. Alveolar epithelial cells from HIV-1 Tg rats had significantly decreased (* P < 0.05) gene expression of both occludin and ZO-1 (Panel A; each value represents the mean ± of 6 determinations). In parallel, alveolar epithelial cells from HIV-1 Tg rats had significantly decreased (* P < 0.05) protein expression of occludin and ZO-1 in both the membrane/cytoskeleton (Triton X-100 insoluble) and the cytosol (Triton X-100 soluble) fractions (Panel B; shown are the summary data expressed as the mean ± SEM of 6 determinations as well as representative immunoblots for each group). Panel C shows representative fluorescent immunocytochemistry images, and illustrates that the relative distribution of ZO-1 and occludin was less uniform in the plasma membranes of alveolar epithelial monolayers derived from HIV-1 Tg rats when compared to monolayers from WT rats, and also showed more granular staining for each protein within the cytoplasm of the cells. These findings were particularly striking for ZO-1 (upper two panels). The magnification bar in the lower right image = 100 microns.

As tight junction proteins mediate the ability of alveolar epithelial cells to regulate paracellular permeability [30], we extended the physiological studies shown in Figure 3 and examined the effects of HIV-1 transgene expression on two key tight junction proteins, namely zonula occludens-1 (ZO-1) and occludin, in the alveolar epithelium. As shown in Figure 4, panel A, gene expression for both ZO-1 and occludin were decreased significantly (P < 0.05), albeit modestly, by alveolar epithelial cells from HIV-1 Tg rats when compared to alveolar epithelial cells from WT rats. These modest inhibitions of gene expression were nevertheless associated with decreases in protein expression. As shown in Figure 4, panel B, both ZO-1 and occludin protein expression were significantly decreased (P < 0.05) in alveolar epithelial cells from HIV-1 Tg rats when compared to alveolar epithelial cells from WT rats, both in the membrane/cytoskeleton (Triton X-100 insoluble) and the cytosol (Triton X-100 soluble) fractions. As paracellular permeability depends not only on tight junction protein expression but also their correct localization within the plasma membrane, we next examined ZO-1 and occludin in alveolar epithelial cell monolayers by fluorescent immunocytochemistry. As shown in the representative images in Figure 4, panel C, the relative distribution of ZO-1 and occludin was less uniform in the plasma membranes of monolayers derived from HIV-1 Tg rats, and also showed more granular staining within the cytoplasm of the cells. These findings were particularly striking for ZO-1 (upper two panels). The magnification bar in the lower right image = 100 microns.

### Treatment of alveolar epithelial cell monolayers from wild type rats with the HIV-related proteins gp120 or Tat *in vitro *altered expression of occludin and ZO-1

**Figure 5:**
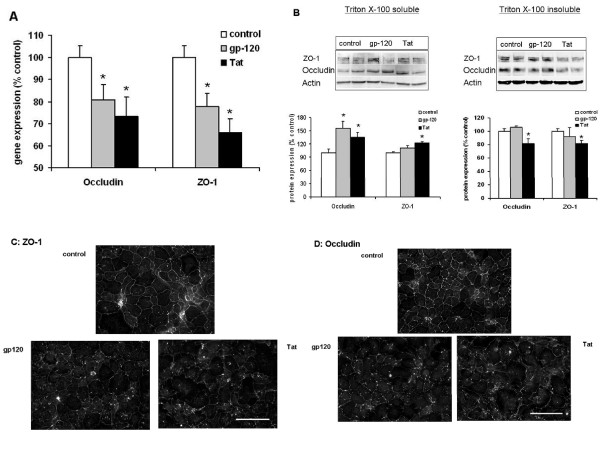
**Treatment of alveolar epithelial monolayers from wild type rats with either gp120 or Tat for 24 hrs altered expression of occludin and ZO-1**. Treatment with either gp120 or Tat significantly decreased (P < 0.05) the gene expression of occludin and Tat when compared to untreated control cells (Panel A). Although occludin and ZO-1 protein expression were either unchanged or increased (* P < 0.05) in the cytosol (Triton X-100 soluble) fractions (Panel B, left side) following these short-term exposures, Tat treatment significantly decreased (*P < 0.05) both occludin and ZO-1 protein expression in the membrane/cytoskeleton (Triton X-100 insoluble) fractions (panel B, right side). In parallel, and consistent with the images shown in Figure 4, treatment with either gp120 or Tat altered the membrane localization of occludin and ZO-1. Shown in panels C and D are representative fluorescent immunocytochemistry images illustrating that the relative distribution of ZO-1 (Panel C) and occludin (Panel D) was less uniform in the plasma membranes of gp120-treated or Tat-treated alveolar epithelial monolayers compared to untreated (control) monolayers, with more granular staining for each protein within the cytoplasm of the cells. The magnification bar in the lower right image of each panel = 100 microns.

To determine whether or not HIV-related proteins, particularly gp120 and Tat, could be implicated more directly in the observed changes in ZO-1 and occludin expression, we exposed alveolar epithelial monolayers from wild type (WT) rats (6–7 days in culture) to recombinant gp120 or Tat for 24 hrs and then assessed the expression of these tight junction proteins. As shown in Figure 5, panel A, treatment with either gp120 or Tat significantly (P < 0.05) decreased both occludin and ZO-1 gene expression by ~20% and 30% respectively compared to no treatment (control). In contrast, treatment with heat-inactivated gp120 had no effect on either occludin or ZO-1 gene expression (not shown). Although this short-term exposure did not reproduce the entire pattern of ZO-1 and occludin protein expression seen in monolayers from HIV-1 Tg rats, Tat treatment decreased (P < 0.05) both ZO-1 and occludin protein levels in the membrane/cytoskeleton (Triton X-100 insoluble) fraction when compared to no treatment (control) as shown in Figure 5, panel B (* P < 0.05 compared to control in each condition). In parallel, immunocytochemical analyses revealed changes in occludin and ZO-1 staining in the plasma membranes of gp120- and Tat-treated monolayers that were similar to those seen in monolayers from HIV-1 Tg rats shown in Figure 4C. Specifically, as shown in the representative images in Figure 5 (panel C shows staining for ZO-1 and panel D shows staining for occludin), treatment with either gp120 or Tat caused some discontinuous staining within the plasma membranes and appeared to increase the amount of punctate staining within the cytosol. The magnification bar in the lower right image of each panel = 100 microns. Therefore, even short-term treatment of naïve alveolar epithelial monolayers with gp120 or Tat *in vitro *produced changes in the expression of ZO-1 and occludin that were consistent with what was observed in the alveolar epithelium of rats that expressed these proteins *in vivo*.

## Discussion

In this study we determined that HIV-1 transgene expression in rats, in which HIV-related proteins including gp120 and Tat are expressed in the absence of viral infection or replication, causes significant oxidant stress and alveolar epithelial barrier dysfunction in the lung. Specifically, the pool of glutathione within the alveolar space of HIV-1 Tg rats was decreased >90%, with a concomitant increase in the ratio of oxidized to reduced glutathione, as well as an increase in tissue levels of hydrogen peroxide. However, despite this significant oxidant stress there was no overt evidence of lung inflammation as reflected by the absence of neutrophil or lymphocyte infiltration of the airways and no change in the levels of several key cytokines. In parallel with the oxidant stress the alveolar epithelial barrier was impaired, with decreased lung liquid clearance *in vivo *that correlated with increased paracellular permeability of alveolar epithelial monolayers *in vitro*. Finally, the expression and membrane localization of two key tight junction proteins, ZO-1 and occludin, were decreased within the alveolar epithelium of HIV-1 Tg rats, consistent with previous findings that Tat alters ZO-1 expression in the blood-brain barrier via redox-sensitive signaling mechanisms [31]. Finally, these effects on ZO-1 and occludin expression could be largely reproduced by treating naïve alveolar epithelial monolayers with gp120 or Tat protein directly *in vitro*. Taken together, these results indicate that HIV-related proteins produce oxidant stress and previously unrecognized barrier dysfunction within the alveolar epithelium, effects that resemble toxicities that have been described in other tissues such as the endothelium of the blood-brain barrier [32,33]. Further, these results suggest a novel mechanism by which HIV infection might render individuals susceptible to acute and chronic forms of respiratory failure. For example, an impaired alveolar epithelial barrier would increase lung edema and worsen gas exchange during acute pneumonia, and could potentially increase the susceptibility to chronic damage from smoking and promote the development of emphysema. When these effects on the alveolar epithelium are coupled with the immune suppression of alveolar macrophages that we recently described in this model [10], the experimental evidence argues that chronic exposure of the alveolar epithelium to HIV-related proteins *in vivo *could contribute to the increased risk of acute and chronic lung disease in HIV-infected individuals.

Many lung disorders are associated with oxidant stress [11], and it is not surprising that HIV infection has long been recognized to cause systemic and pulmonary oxidant stress, including significant lowering of glutathione levels [12-15]. Our findings in this study provide compelling evidence that HIV-related proteins exert oxidant stress within the alveolar space independently of active viral replication. Further, as the levels of glutathione were decreased by ~90% in the lung lavage fluid (which largely reflects the alveolar epithelial lining fluid), these results suggest that the alveolar compartment is particularly vulnerable to oxidant damage during HIV infection. The investigation of the mechanisms by which these HIV-related proteins induce oxidant stress within the alveolar space was beyond the scope of this initial study. Whether or not putative mechanisms that have been implicated with individual HIV-related proteins such as gp120 or Tat in cell culture studies *in vitro *are involved in the alveolar space *in vivo *is at present unknown. However, these findings suggest the possibility that therapeutic strategies designed to augment glutathione pools within the airway could mitigate the effects of chronic HIV infection.

The potential for complementing current HAART strategies with antioxidant supplements is further supported by emerging evidence that the oxidant stress caused by chronic HIV infection could be exacerbated by other factors such as alcohol abuse, which is a common co-morbid condition in this population [34,35]. Our group has used both animal models and clinical studies to demonstrate that chronic alcohol abuse also causes oxidant stress within the alveolar space [24,26], as well as alveolar epithelial [24,36,37] and macrophage defects [9,38-40] that are remarkably similar to what we have identified in the HIV-1 transgenic rat model, including altered expression and membrane localization of tight junction proteins [41]. Therefore, chronic alcohol ingestion could exacerbate HIV-mediated oxidant stress as well as epithelial and endothelial barrier dysfunction, and could thereby account for the poorer outcomes in these individuals [34]. It can be difficult to extrapolate from animal models and separate association from causation in complex human diseases, and to date the evidence supporting the clinical use of anti-oxidants as a treatment for a specific lung disease is lacking. However, the combination of HIV infection and chronic alcohol abuse may represent a uniquely vulnerable population that could respond to dietary supplements with glutathione precursors, such as S-adenosylmethionine. In addition, we recently determined that HIV-1 transgene expression impairs zinc homeostasis within the alveolar space, and that dietary zinc supplementation improves alveolar macrophage immune function [10].

There are limitations in this initial study. We did not examine other potential contributors to the oxidant stress such as alterations in nitric oxide balance ("nitrosative stress"). In addition, our evaluation of potential dysregulation of inflammation within the alveolar space was restricted to quantification of inflammatory cells and three key cytokines; although we found no gross evidence of inflammation in the airways of HIV-1 transgenic rats, we clearly did not exclude important changes such as alterations in lymphocyte subsets or other perturbations. In light of the significant dampening of alveolar macrophage immune function that we previously identified in this model [10], one might expect to see impaired recruitment of neutrophils and/or appropriate lymphocyte subsets during stresses such as pneumonia. Another important limitation of this study is that at present we cannot conclude that the effects of chronic exposure to HIV-1-related proteins in the rat lung mirror the effects in the lungs of HIV-infected humans. Importantly, whether or not human or non-human cells are used to study the effects of these proteins on cells *in vitro*, it remains problematic to extend such findings to the clinical setting. Therefore, our findings in this transgenic rat model can serve as a guide in designing clinical studies but must be confirmed in the human context. Although these experimental findings need to be translated to the clinical setting, it is compelling to consider that glutathione precursors and zinc could be adjunctive therapies in the chronic treatment regimens for HIV infection. Such strategies could be applied fairly easily to determine if the alveolar macrophage dysfunction we identified in this experimental model [10] does indeed translate to the clinical setting. For example, if dietary zinc and/or glutathione supplements improved alveolar macrophage function in otherwise healthy HIV-infected individuals (it is not feasible to obtain sufficient quantities of alveolar epithelial cells), this would provide additional validation of the transgenic rat model and more importantly would provide the rationale for a multi-center interventional trial.

## Conclusion

In summary, we report that HIV-1 transgene expression causes previously unrecognized oxidant stress within the alveolar space, and that this oxidant stress is associated with changes in tight junction protein expression and barrier dysfunction in the alveolar epithelium even in the absence of any obvious airway inflammation. Although previous studies have determined that HIV-related proteins induce oxidant stress and disrupt the blood-brain endothelial barrier with significant consequences to the central nervous system [19,33,42], to our knowledge there are no studies to date that demonstrate comparable toxicities within the alveolar epithelium. These findings are provocative in that they suggest a novel mechanism by which HIV infection renders individuals susceptible to acute and chronic forms of lung injury, and in particular could help explain how HIV infection increases the risk of putatively non-infectious lung diseases such as emphysema [3-5,43]. Finally, this study and recent work from our laboratory [10,44] suggest that HIV-1 transgenic rodent models offer unique opportunities to determine the pathological effects of HIV-related proteins independently of viral infection and/or replication, and provide powerful tools to perform pre-clinical assessment of complementary therapies such as glutathione and/or zinc supplementation.

## Methods

### Animals

Male HIV-1 transgenic Fischer 344 rats and Fischer 344 wild type rats were purchased from Harlan (Indianapolis, Indiana) and bred in the animal facility at the Atlanta VA under a 12:12 light-dark cycle. The transgenic rat is hemizygous NL4-3Δgag/pol, in which the 3' region of *gag *and the 5' region of *pol *is deleted [23]. Therefore, breeding pairs produce off-spring that are ~50% HIV-1 transgenic and ~50 wild type (confirmed by genotyping), allowing the use of littermate wild type animals as controls. The HIV-1 transgenic rats have dense cataracts at birth but otherwise appear healthy and develop normally; however, by 6 months of age they begin to display evidence of systemic disease including poor weight gain and muscle atrophy that progress over time. In these studies, we used HIV-1 transgenic rats and their wild type littermates between the ages of 7 and 9 months. Food and water were provided *ad libitum*. All procedures were approved by the Atlanta Veteran Affairs Medical Center Institutional Animal Care and Use Committee.

### Analyses of lung lavage fluid for total and differential cell counts, cytokine levels, and glutathione (GSH) and glutathione disulfide (GSSG) determinations

Bronchoalveolar lavage (BAL) fluid samples (n = 4) were assessed for 1) cell counts using a standard hemocytometer (total counts) and manual counting of cells stained with Wright's stain and analyzed under low power with a light microscope, 2) levels of IL-2, TNFα, and IL-4 using a Luminex assay (Linco Research Inc., MO) following the manufacturer's instructions, and 3) GSH and GSSG levels by derivatizing the fluid with iodoacetic acid and dansyl chloride and analyzing by HPLC with fluorescence detection [45]. GSH and its dominant oxidized form glutathione disulfide (GSSG) were quantified by integration relative to an internal standard [45]. The levels of GSH (the predominant component of the redox couple under normal conditions) were expressed in mM concentrations in the lavage fluids. In parallel, the relative concentrations of GSSG to GSH in the lavage fluids were expressed as the GSSG:GSH ratios, which is a standard method of assessing oxidation of the GSH redox couple in biological fluids [45].

### Lung tissue hydrogen peroxide analysis

As previously described [44], lung tissue (n = 6) was isolated and hydrogen peroxide was quantified using the Amplex Red reagent (Molecular Probes, OR), a highly sensitive and stable probe utilized as a fluorogenic substrate for horseradish peroxidase. Briefly, lung tissue was isolated and incubated at 37°C for 30 minutes in solution containing Amplex Red reagent, horseradish peroxidase, and a buffer solution. Supernatant was then collected, and fluorescence was read at 560 nm. Concentrations were determined using extrapolation of a standard curve.

### Lung liquid clearance *in vivo*

We used a method we have published previously [27,28]. Rats were anesthetized with sodium pentobarbital and a tracheostomy cannula was introduced. A saline solution (2 mL) was introduced into the trachea, and the rats were mechanically ventilated for 30 minutes. Ventilation was performed using a Harvard rodent ventilator with a tidal volume of 2.5 mL (~8 mL/kg body weight) at a rate of 60 breaths/min for 30 minutes. After 30 minutes of ventilation, a median sternotomy was performed, and the right lung was excised and weighed to obtain the wet weight. The lung was then dehydrated overnight at 70°C and the dry weight was then determined. The ratio of the wet weight to dry weight was calculated and expressed for each lung sample.

### Alveolar epithelial monolayer culture and treatments

Alveolar epithelial type II cells (AEC) were isolated from HIV-1 transgenic and control rats using a previous protocol [46]. Briefly, rats were anesthetized, and a tracheostomy was placed followed. The lungs were perfused blood-free with saline via the pulmonary artery and after *en bloc *isolation were filled via the tracheostomy cannula with a solution containing porcine pancreatic elastase. The lung parenchyma was then isolated from the large airways and minced, and then successively filtered through 100- and 20-μM nylon mesh. The recovered cells were plated onto plastic dishes pre-coated IgG to remove the alveolar macrophages and other immune cells. After 1 h of incubation at 37°C, the non-adherent cells were gently aspirated from the plates. Cells obtained by this method contained ~90% AEC that are >90% viable by trypan blue exclusion. Freshly isolated AEC were then re-suspended. In selected experiments, cells were assessed for the expression of the HIV-1-related proteins gp120 and Tat by ELISA and western blot analysis, respectively. Cells were plated using 300,000 cells per ml onto 12 mm diameter permeable membranes (0.4 μM pore, Corning) and cultured for a total of 8 days at 37°C in 90% air-10% CO_2_. The medium was changed every 48 hours. To determine the independent and direct effects of HIV-1-related proteins on alveolar epithelial tight junction proteins, 6 day AEC monolayer cultures from wild type rats were treated with either gp120 (100 ng/ml, ImmunoDiagnostics, MA) or Tat (1 μg/ml, ImmunoDiagnostics, MA) in serum free medium for 24 hrs at 37°C, and then ZO-1 and occludin mRNA and protein expression were assessed by real-time PCR and western blot analyses, respectively. In parallel, the specificity of gp120-induced effects was also assessed by treating some monolayers with heat-inactivated gp120 (incubated at 85°C for 30 min).

### Alveolar epithelial permeability assay

The barrier function of the cell monolayers after 7 days in culture was determined as we have done previously [36] by determining the percent flux of ^3^H-labeled sucrose across AEC monolayers cultured in transwells which contained the media covering the apical surfaces of the cultured cells. Permeability was defined as the fraction of the initial radioactivity placed on the apical surface that appeared on the basolateral surface of the monolayer after 2 hours, and expressed as a percentage. We have previously shown that this method correlates well with radiolabeled inulin flux across these monolayers, and with the flux of radiolabeled albumin flux across the alveolar epithelial barrier in *vivo *[36].

### Western analyses

Cultured AEC were washed with cold PBS, lysed in Triton X-100 buffer (25 mM Hepes/NaOH (pH7.4), 150 mM NaCl, 4 mM Na_2_EDTA, 25 mMNaF. 1%Triton X-100, 1 mM Na_3_VO_4_, 1 mM PMSF and protease inhibitor cocktail (Roche)) on ice for 30 min and then centrifuged at 14,000 g for 10 min. The supernatants were collected as the Triton X-100 soluble fraction represented cytosolic fraction. The pellets were re-suspended in SDS lysis buffer (25 mM HEPES/NaOH (pH7.4), 4 mM Na_2_EDTA, 25 mM NaF. 1%Triton X-100, 1 mM Na_3_VO_4_), sonicated, boiled for 5 min and centrifuged for 10 min at 14,000 g. The supernatant was used as the Triton X-100 insoluble fraction represented most membrane/cytoskeleton fraction [47,48]. The protein concentration was determined by the BCA method. An equal amount of protein (15–50 μg) was separated on 8% SDS polyacrylamide gel and transferred to PVDF membrane. After blocking with 5% milk/TBST, the membrane was incubated with anti-zonula occludens-1 (ZO-1) or anti-occludin antibody (Invitrogen) overnight and then corresponding HRP-conjugated 2nd antibody was added prior to ECL plus substrate (GE healthcare). The immunoreactive bands were captured with ChemiDoc XRS system (Bio-Rad). Same membrane was stripped and incubated for anti-actin antibody (Santa Cruz biotechnology, Inc., Santa Cruz).

### Immunocytofluorescence

Freshly isolated AEC were cultured on glass coverslips for 7 days as we have published previously [41]. Briefly, the monolayers were washed with PBS, fixed and permeabilized with methanol/acetone 1:1 for 2 min, and then washed with PBS + 0.5% Triton X-100 (PBS/TX) and incubated with PBS + 0.5% Triton X-100 + 2% goat serum (PBS/TX/GS). The primary antibody was diluted in PBS + 2% goat serum (PBS/GS) and then added on cell layers for 1 hr at room temperature prior incubated with Cy2-conjugated goat anti-rabbit IgG. The images were captured by using an Olympus IX-70 inverted fluorescence microscope outfitted with a Hamamatzu Orca charge-coupled device and then analyzed with Image Pro software.

### Statistics

One-way analyses of variance were performed followed by Student-Newman-Keuls post-hoc tests using SigmaStat v2.0 software or student T-test by Prism (GraphPad, San Diego, CA). Significance was accepted at P < 0.05.

## Competing interests

The authors declare that they have no competing interests.

## Authors' contributions

CL supervised the HIV-1 transgenic rat colony, performed the lung liquid clearance measurements (Figure 3A), isolated lung tissue, alveolar epithelial cells, and lung fluid (necessary for the findings in all of the figures), and wrote the initial draft of the manuscript. XF determined tight junction (occludin and ZO-1) gene and protein expression (Figures 4 and 5). PCJ determined alveolar epithelial cell permeability *in vitro *(Figure 3B). BAJ, RLS, and DPJ performed the studies that quantified oxidant stress in the HIV-1 transgenic rats (Figures 1 and 2). MK performed the immunocytochemistry for occludin and ZO-1 protein localization in alveolar epithelial monolayers *in vitro *(Figures 4 and 5). DMG designed the experiments and analyzed the data with CL (who was a post-doctoral research fellow in DMG's laboratory), supervised the overall experimental plans, and revised and edited the final manuscript.
